# Dissociation and flow cytometric isolation of murine intestinal epithelial cells for multi-omic profiling

**DOI:** 10.1016/j.xpro.2022.101936

**Published:** 2022-12-13

**Authors:** Yong Ge, Mojgan Zadeh, Mansour Mohamadzadeh

**Affiliations:** 1 Department of Microbiology, Immunology & Molecular Genetics, University of Texas Health, San Antonio, TX, USA; 2 Division of Gastroenterology & Nutrition, Department of Medicine, University of Texas Health, San Antonio, TX, USA

**Keywords:** Cell isolation, Flow Cytometry/Mass Cytometry, Sequencing, Single Cell

## Abstract

Intestinal epithelium is composed of several cell types, which can be dissociated but difficult to maintain high cell viability due to anoikis. Herein, we describe a step-by-step protocol for the isolation of highly viable intestinal epithelial cells using ethylenediaminetetraacetate acid and TrypLE Express, which can subsequently be employed for multi-omic analyses, including single-cell RNA sequencing.

For complete details on the use and execution of this protocol, please refer to Ge et al. (2022).^1^

## Highlights

• Dissociation of murine ECs from intestine
• We describe the procedures to stain and analyze ECs by flow cytometry
• Purified ECs can be used for multi-omic analysis, including scRNA-seq

## Abstract (editor highlights)

Publisher’s note: Undertaking any experimental protocol requires adherence to local institutional guidelines for laboratory safety and ethics.

## Graphical abstract

**Figure:**
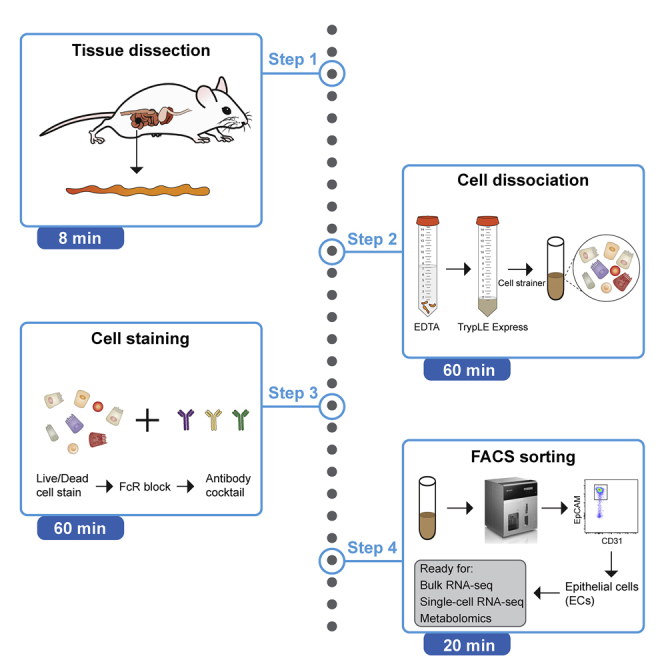

## Before you begin

Our protocol describes in detail the required steps for the isolation and analysis of epithelial cells derived from mouse distal small intestine (ileum). The same protocol can also be used for isolating cecal and colonic epithelial cells. The protocol focuses on the transcriptomic, and metabolomic analysis of ileal epithelial cells at homeostasis, but we have also used it to describe changes in epithelial cell transcriptome and metabolome following intestinal *Salmonella* Typhimurium infection.

1. Animal studies must be approved by an Institutional Animal Care and Use Committee (IACUC) and performed in accordance with IACUC guidelines.
2. Mouse strain selection will depend on the experiment. We have extensively analyzed 1–12-week-old C57BL/6J mice.
3. Prepare solutions in a biosafety cabinet and store them at 4°C or on ice prior to procedures (15 min; refer to materials and equipment for recipe tables).
  a. EDTA buffer.
  b. FACS buffer.
  c. MACS buffer.
  d. Post-sort buffer.
4. Equilibrate a centrifuge compatible with 15 mL and 50 mL falcon tubes to 4°C.

## Key resources table

**Table:** 

REAGENT or RESOURCE	SOURCE	IDENTIFIER
**Antibodies**		

APC anti-mouse CD45	BioLegend	Cat# 103112, RRID: AB_312977
FITC anti-mouse CD31	BioLegend	Cat# 102406, RRID: AB_312901
PE/Cyanine7 anti-mouse TER-119	BioLegend	Cat# 116222, RRID: AB_2281408
PE anti-mouse CD326 (EpCAM)	Thermo Fisher Scientific	Cat# 12-5791-83, RRID: AB_953617

**Chemicals, peptides, and recombinant proteins**		

Dulbecco’s phosphate buffered saline (DPBS)	Genesee Scientific	Cat# 25-508
Ethylenediaminetetraacetate acid (EDTA)	Thermo Fisher Scientific	Cat# BP2482100
TrypLE express enzyme	Thermo Fisher Scientific	Cat# 12604021
FcR blocking reagent	Miltenyi Biotec	Cat# 130-092-575
Bovine serum albumin (BSA)	Sigma-Aldrich	Cat# A3059
β-Mercaptoethanol	Sigma-Aldrich	Cat# 444203
RPMI-1640 medium	Corning	Cat# 10041CV
Fetal bovine serum (FBS)	Gibco	Cat# 16000-044
ViaStain AOPI staining solution	Nexelcom	Cat# CS2-0106

**Critical commercial assays**		

LIVE/DEAD Fixable Violet Dead Cell Stain Kit	Thermo Fisher Scientific	Cat# L34964
RNeasy Plus Micro Kit	Qiagen	Cat# 74034

**Experimental models: Organisms/strains**		

C56BL/6J mice	Jackson Laboratory	#000664

**Software and algorithms**		

FlowJo v10	Tree Star	N/A

**Other**		

Cell strainer (70 μm)	Genesee Scientific	Cat# 25-376
FACS Tube with w/ 2-Position Cap	Genesee Scientific	Cat# 28-150
FACS Tubes w/ 35 μm Strainer Cap	Genesee Scientific	Cat# 28-154
Cellometer Auto 2000	Nexcelom	N/A
SH800S Cell Sorter	Sony	N/A
CytoFlex SRT Cell Sorter	Beckman Coulter	N/A
Animal feeding needle	Thermo Fisher Scientific	Cat# 01-290-11B

## Materials and equipment

**Table:** EDTA buffer

Reagent	Final concentration	Amount
EDTA (0.5 M)	30 mM	30 mL
DPBS	N/A	470 mL
Total	N/A	500 mL

Store at 4°C for up to 1 month.

***Note:*** Use magnesium- and calcium-free DPBS.

***Alternatives:*** PBS can be used to replace DPBS in this protocol.

**Table:** FACS buffer

Reagent	Final concentration	Amount
EDTA (0.5 M)	1 mM	1 mL
BSA	0.5%	2.5 g
DPBS	N/A	499 mL
Total	N/A	500 mL

Store at 4°C for up to 1 month.

**CRITICAL:** Do not add sodium azide into FACS buffer as it may reduce cell viability and inhibit cell metabolic activity.

**Table:** MACS buffer

Reagent	Final concentration	Amount
EDTA (0.5 M)	2 mM	0.2 mL
FBS	2%	1.0 mL
DPBS	N/A	48.8 mL
Total	N/A	50 mL

Store at 4°C for up to 1 week.

**Table:** Post-sort buffer

Reagent	Final concentration	Amount
FBS	20%	10 mL
RPMI-1640 medium	N/A	40 mL
Total	N/A	50 mL

Store at 4°C or on ice and use on the same day.

## Step-by-step method details

### Collection of intestinal tissues

**Timing: 8 min**

1. Euthanize mouse by cervical dislocation or other approved method and expose the abdominal cavity.
  a. Lay euthanized mouse on its back on a hard surface (e.g., Rodent Surgery Board).
  b. Lift the skin of the lower abdomen with tweezers and make a V-shape incision with scissors;
  c. Cut away skin to expose abdominal cavity.
2. Collect ileum tissues.
  a. Cut the colon from the anus and gently pull out the intestine from the abdominal cavity.
  b. Cut the end of the small intestine from the cecum.
  c. Take the similar length as colon moving proximal from the ileocecal junction (ileum).
3. Remove the fat and mesentery tissues from dissected ileum with tweezers; Flush away the luminal contents using cold DPBS with an animal feeding needle.
4. Using scissors, cut open the ileum longitudinally. Place tissues in 50 mL falcon tube pre-filled with 10 mL cold DPBS and keep the tube on ice until all animals are processed.

**CRITICAL:** After tissue dissection, perform all procedures as fast as possible and keep all buffers and preparations at 4°C or on ice unless specified otherwise.

### Preparation of epithelial single-cell suspension

**Timing: 60 min**

5. Wash the tissue three times with cold DPBS.
  a. Remove DPBS by pouring it out.
  b. Add 10 mL of cold DPBS and shake the tube up and down for 20–30 s.
  c. Repeat steps 6a and 6b two more times.
6. Transfer tissue to a petri dish and slice into small fragments roughly 2–3 cm in length.
7. Transfer tissue fragments into 15 mL falcon tube pre-filled with 7 mL of cold EDTA buffer and keep the tube on ice.
  a. Shake the tube by hand (up and down for about 60 cycles; 0.5 s/cycle) every 7–8 min.
  b. After 30 min of ice incubation, shake the tube vigorously for 2 min.
  c. Use a tweezer to remove the tissues from the tube.
8. Centrifuge at 500 *g* and 4°C for 5 min; remove supernatant and add 10 mL of cold DPBS to wash cell pellets.
9. Pellet the cells and add TrypLE Express Enzyme to the tube.
  a. Spin down the cells at 500 *g* and 4°C for 5 min.
  b. Remove the supernatant by pouring. Using pipette tips, remove the residual supernatant.
  c. Immediately add 2 mL TrypLE Express Enzyme and gently resuspend cell pellets using 1 mL pipette tips.
  d. Keep at 22°C–25°C for 10 min; During incubation, pipette up and down 10 times with 1 mL pipette tips to gently break cell aggregates every 3–4 min.

***Note:*** Room-temperature (22°C–25°C) incubation is recommended in this step as higher temperature (37°C) may impact cell viability.

10. Fill the tube with 10 mL of cold DPBS and pass through 70 μm cell strainer placed on a 50 mL falcon tube; add another 10 mL of cold DPBS to wash the cell strainer; collect all the flow-through (single-cell suspension).
11. Centrifuge at 500 *g* and 4°C for 5 min. Cell pellets are ready for staining.

### Procedures for cell surface staining

**Timing: 60 min**

12. Resuspend cells using 1 mL of Live/Dead staining solution (1:1000 dilution in DPBS); transfer to FACS tubes and keep in a 4°C fridge for 20 min.
13. Wash cells by directly adding 4 mL of cold DPBS to the tube; spin down to remove supernatant.
14. Add 300 μL of FcR blocking reagent (1:50 dilution in FACS buffer) to resuspend cell pellets; keep in a 4°C fridge for 5 min.
15. Dilute antibodies (anti-CD45, anti-CD31, anti-TER-119 and anti-EpCAM) into FACS buffer (1:50 dilution). Without wash, directly add 300 μL of antibody cocktail to the FACS tube (1:100 final dilution); gently vortex to mix; keep in a 4°C fridge for 30 min.
16. Wash cells by adding 4 mL of cold FACS buffer; spin down the cells at 500 *g* and 4°C for 5 min; remove supernatant by pouring out the buffer.
17. Resuspend cells in 300 μL of MACS buffer and transfer to FACS tubes mounted with 35 μm cell strainer cap; Keep cells on ice.

***Note:*** If cells do not pass through cell strainer by gravity, a short centrifugation step can be applied.

### Flow cytometry and cell sorting

**Timing: (20 min per sample)**

18. Acquire samples on FACS machine and sort cells into post-sort buffer.

***Note:*** For our studies, we used SH800S cell sorter (Sony, Japan) and CytoFlex SRT cell sorter (Beckman Coulter, USA) and analyzed the data using FlowJo software package (Tree Star, USA).

19. Mix cells with AOPI staining solution at 1:1 ratio; Load 20 μL onto slide and count using Auto 2000. Adjust volume to obtain the required cell recovery target.

***Alternatives:*** Cells can also be counted using a hemocytometer.

***Note:*** The actual number of cells is about 65%–80% of counts recorded by cell sorter.

20. After cell counting, immediately proceed to downstream analysis:
  a. If cells are used for bulk RNA-seq, spin down the cells at 500 *g* and 4°C for 5 min and carefully remove supernatant by pipetting; resuspend cell pellets in 350 μL of Buffer RLT Plus supplemented with 1% β-mercaptoethanol.
    **Pause point:** samples can be stored at −80°C for months until being further processed for RNA isolation using RNeasy Micro Plus kit (https://www.qiagen.com/us/products/discovery-and-translational-research/dna-rna-purification/rna-purification/total-rna/rneasy-plus-kits/).
    **CRITICAL:** Removal of supernatants as much as possible is crucial for ensuring efficient cell lysis. We normally keep less than 20 μL of supernatants in the tube so that the cell pellets are not disturbed.
    **CRITICAL:** Wear a mask when handling β-mercaptoethanol.
  b. If cells are used for single-cell RNA-seq, immediately proceed to GEM generation and barcoding (https://www.10xgenomics.com/support/single-cell-immune-profiling/documentation/steps/library-prep/chromium-single-cell-5-reagent-kits-user-guide-v-2-chemistry-dual-index).
  c. If cells are used for metabolomic analysis, spin down the cells at 500 *g* and 4°C for 5 min and carefully remove supernatant by pipetting; resuspend cell pellets in1 mL cold DPBS and transfer to 1.5 mL Eppendorf tube for washing. Pellet the cells by centrifugation at 500 *g* and 4°C for 5 min; completely remove the supernatants.
    **Pause point:** Cells can be snap-frozen in liquid nitrogen or on dry ice and store at −80°C for months until metabolite extraction.

## Expected outcomes

**Figure 1:**
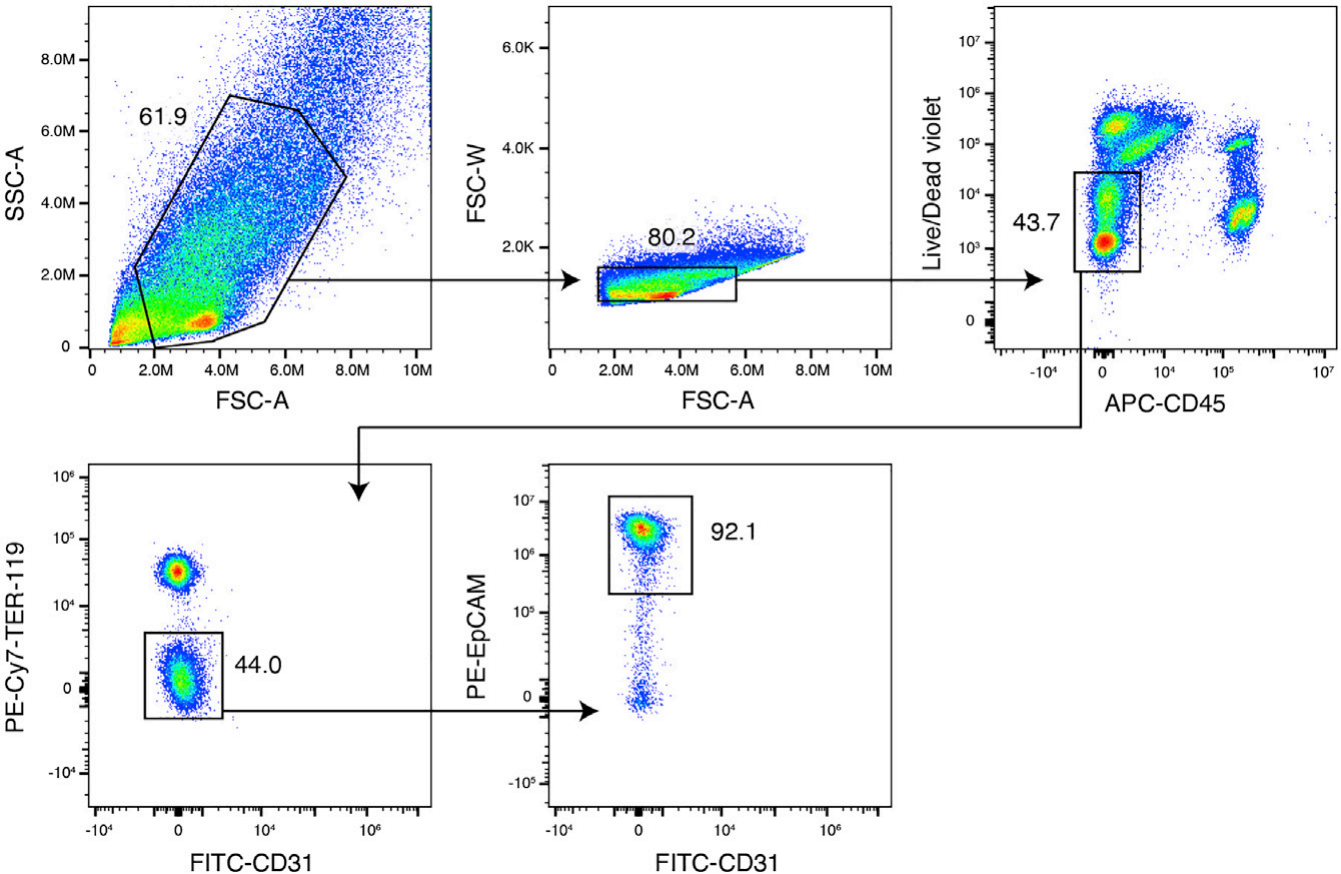
Flow cytometry analysis of epithelial cells isolated from mouse ileum After doublet cell and dead cell exclusion, the intestinal epithelial cells are defined as CD45^-^ TER-119^-^ CD31^-^ and EpCAM^+^ cell population.

This protocol allows for successful isolation of all expected intestinal epithelial cell types.^2^ For sample plots of flow cytometry analysis see Figure 1.

**Figure 2:**
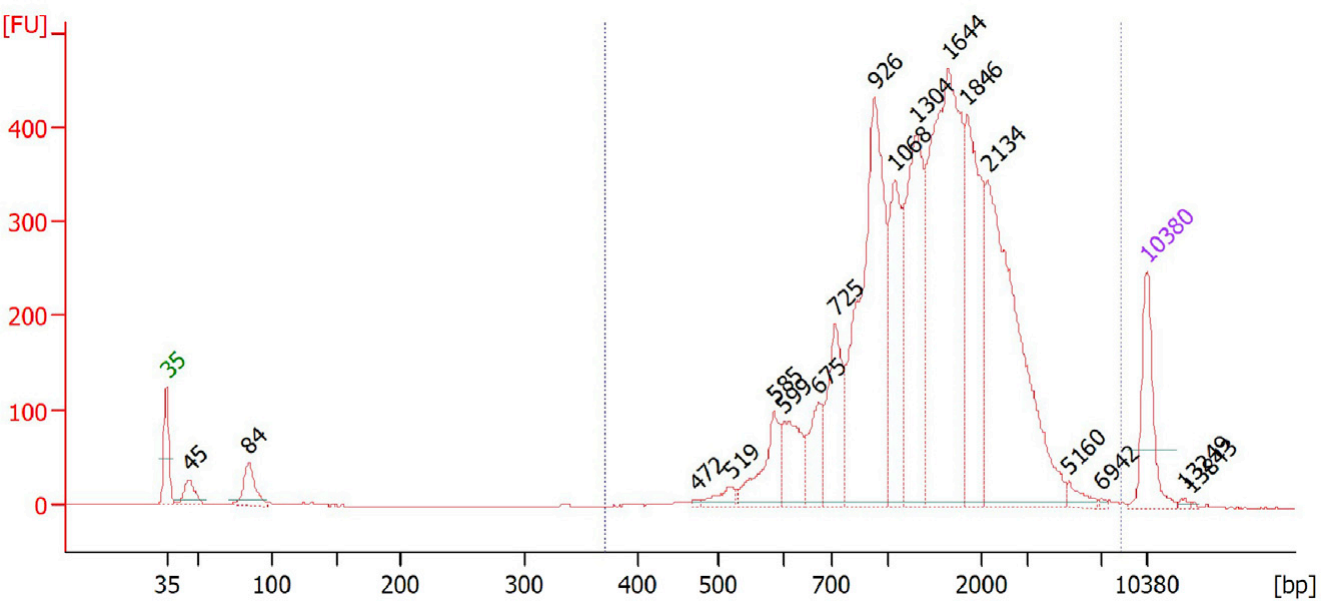
Bioanalyzer analysis of cDNA generated from ileal epithelial cells

Successful dissociation and cell sorting should yield a viability of 75% or greater for optimal downstream application results. The cell number is dependent on the size and health status of the tissue. The ileum of healthy mice (8 weeks old) will typically yield > 200K cells. For single cell sequencing, the cDNA should yield a distinct peak between 400 bp and 10 kb (Figure 2).

## Limitations

This protocol was only tested on mouse ileum, cecum and colon and may need to be adapted for other mucosal sites.

Red blood cell lysis may lead to a significant reduction in cell viability and/or count.

Insufficient dissociation during TrypLE digestion (e.g., without pipetting up and down) may result in lower cell count.

## Troubleshooting

### Problem 1

Insufficient cell dissociation.

### Potential solution

Before adding TrypLE Express, remove leftover supernatants by pipetting as dilution of TrypLE Express reduces its enzymatic activity. During incubation with TrypLE Express, make sure to pipette up and down every 3–4 min to break intestinal crypts. The digestion mixture should be homogeneous with no visible crypts after 10 min incubation.

### Problem 2

Loss of cell pellets after TrypLE Express digestion (step 12).

### Potential solution

Cell pellet may be loose in 50 mL falcon tube after centrifugation. Always check the cell pellets and gently pour the supernatants out.

### Problem 3

High red blood cell number in the single cell resuspension.

### Potential solution

Remove mesentery tissues and blood vessels from the intestine as much as possible. Blood removal from anesthetized mouse via heart-puncture could help to reduce red blood cells. Make sure to include anti-mouse TER-119 antibody to exclude red blood cells during cell sorting.

### Problem 4

Low yield during cell sorting.

### Potential solution

Ensure the antibody dilutions and gating strategy.

### Problem 5

Low cell viability after FACS sorting (<75%).

### Potential solution

Timely processing of tissue after dissection: Complete the tissue processing procedure as soon as possible following tissue collection. Except for TrypLE enzymatic digestion, keep all buffers and samples at 4°C or on ice as indicated. Sort cells right after cell staining and make sure to maintain the sample chamber and collection area at 4°C during cell sorting.

## Resource availability

### Lead contact

Further information and requests for resources and reagents should be directed to and will be fulfilled by the lead contact, Mansour Mohamadzadeh (Zadehm@uthscsa.edu).

### Materials availability

This study did not generate new unique reagents.

## Data Availability

This study did not generate new data or code.
